# Intravascular Imaging Guidance for Left Main Interventions: The Emerging Role of Optical Coherence Tomography

**DOI:** 10.3390/jcdd12120497

**Published:** 2025-12-17

**Authors:** Antonios Rigas Papapanagiotou, Antonios Karanasos, Athanasios Papageorgiou, Michail I. Papafaklis, Athanasios Moulias, Grigorios Tsigkas, Periklis Davlouros

**Affiliations:** Department of Cardiology, University of Patras, 26504 Patras, Greece

**Keywords:** left main coronary artery disease, unprotected left main, percutaneous coronary intervention, intravascular imaging, optical coherence tomography, intravascular ultrasound, stent optimization, bifurcation lesions, plaque morphology

## Abstract

Left main (LM) coronary artery disease remains a critical and high-risk clinical entity with considerable prognostic impact. While surgical revascularization has long been the standard of care, advances in percutaneous coronary intervention (PCI) techniques have significantly improved outcomes, challenging traditional treatment paradigms. Nevertheless, PCI in LM lesions continues to be associated with increased rates of repeat revascularization. This has underscored the importance of precise procedural planning and stent optimization, for which intravascular imaging is central. Among available modalities, intravascular ultrasound (IVUS) is well-established and widely endorsed in clinical guidelines for LM PCI. Optical coherence tomography (OCT), although increasingly utilized in other coronary settings, has a more limited but growing body of evidence in LM disease. This review explores the evolving application of OCT in LM interventions, focusing on its capabilities in plaque characterization, vessel sizing, stent selection, and identification of failure mechanisms such as malapposition and underexpansion. In addition, it discusses the utility of OCT in guiding bifurcation strategies and provides a comparative assessment with IVUS, integrating the most recent clinical data.

## 1. Introduction

Left main (LM) coronary artery disease (CAD) is one of the most critical and prognostically significant forms of coronary pathology, owing to the extensive myocardial territory at risk. Continuous advancements in interventional cardiology, including refinements in devices and procedural strategies, have progressively redefined the therapeutic landscape of LM disease. Notably, the routine utilization of second-generation drug-eluting stents (DES) has markedly reduced the historical disparity between percutaneous coronary intervention (PCI) and coronary artery bypass grafting (CABG), with contemporary data demonstrating comparable long-term survival among patients with LM disease and low-to-intermediate SYNTAX scores, although CABG maintains a superior overall clinical benefit.

Given the anatomical complexity and prognostic impact of LM lesions, current European guidelines endorse the use of intravascular imaging (IVI) guidance, by either intravascular ultrasound (IVUS) or optical coherence tomography (OCT), to improve outcomes in LM PCI. Specifically, IVUS has traditionally been used for guidance in these procedures, having also an established role in assessing lesion significance in LM lesions [1,2,3]. OCT is an emerging high-resolution imaging modality that has been used for procedural guidance in coronary interventions, showing improved procedural outcomes, especially in complex lesions, with increasing utilization in LM interventions. The aim of the current document is to provide a comprehensive review of the use of OCT for the guidance of left main interventions.

## 2. The Role of Intravascular Imaging in Guiding PCI

Although coronary angiography has traditionally been used for procedural guidance in PCI, it is associated with inherent limitations in evaluating lesion severity, disease burden, and plaque composition. Moreover, its diagnostic accuracy is subject to significant interobserver variability [4]. Intravascular imaging techniques, such as IVUS and OCT, may overcome such shortcomings. By providing high-resolution tomographic cross-sections of the coronary vasculature, these modalities offer enhanced insights into the pathophysiology of atherosclerotic disease, support informed decision-making during catheterization procedures, facilitate the selection of optimal interventional strategies, and ultimately contribute to the procedural success and durability of PCI [5].

### 2.1. Intravascular Ultrasound (IVUS)

IVUS has been shown to be an important tool in improving the outcome of PCI in both clinical trials [6,7], and meta-analyses [8]. IVUS-guided PCI had a significantly lower rate of major adverse cardiac events (MACE), target vessel failure (TVF) and stent thrombosis (ST) compared to angiographic guidance at long-term follow-up [6,7,9]. Current ESC/EACTS guideline recommendations reflect these data, giving IVI, i.e., IVUS or OCT, a class IA recommendation for procedural guidance in complex lesions, including left main lesions [2]. In this context, the use of IVUS in LM disease is strongly advocated for multiple purposes, including the evaluation of angiographically ambiguous LM lesions, selection of lesion preparation strategy, stent sizing, stent optimization post-implantation, and investigation of stent failure mechanisms.

#### 2.1.1. Assessment of LM Lesion Severity by IVUS

IVUS enables comprehensive 360-degree tomographic imaging of the coronary vessel, allowing for precise measurement of the minimum lumen area (MLA) and objective quantification of stenosis severity. An MLA threshold of 6.0 mm^2^ has been shown to adequately discriminate patients in need of revascularization, with lesions with MLA lower than 6.0 mm^2^ associated with a worse prognosis if untreated [3,10]. It should be however noted that the accuracy of these measurements may be affected by population-specific variations, as shown in a South Korean study that proposed that a lower MLA cut-off value of 4.5 mm^2^ may be more appropriate in Asian populations [11]. The application of IVUS in the evaluation of LM lesions may be technically challenging in specific scenarios, such as in cases of short LM, eccentric plaque morphology, diffuse CAD, heavy calcification, vessel overlap, or catheter-induced spasm [9]. Consequently, a more comprehensive lesion assessment may necessitate adjunctive evaluation, usually by fractional flow reserve (FFR), a modality that can evaluate the physiological significance of LM lesions [12]. A FFR value of less than 0.80 is generally indicative of a hemodynamically significant stenosis also in LM lesions, often correlating well with IVUS findings, as demonstrated by Kang et al. [13].

#### 2.1.2. Studies of IVUS-Guided LM PCI

In dedicated LM cohorts, IVUS guidance has shown consistent clinical benefit, while concrete optimization targets have been identified. The MAIN-COMPARE study was an observational study of PCI versus CABG for the treatment of left main disease [9]. A sub-analysis of patients with LM disease undergoing PCI (*n* = 975), showed that 10-year death was numerically lower with IVUS versus angiography (16.4% versus 31.0%; adjusted HR for death 0.75, 95% CI 0.55–1.03) and that, in propensity–matched pairs, the composite of death/Q-wave MI/stroke was significantly lower (HR 0.71, 95% CI 0.52–0.97) [9]. A nationwide registry analysis from the SCAAR registry (*n* = 2468) found that IVUS use independently reduced all-cause death/restenosis/definite stent thrombosis (HR 0.65, 95% CI 0.50–0.84) and all-cause death (HR 0.62, 95% CI 0.47–0.82) [14]. In the NOBLE PCI arm substudy (IVUS use in 72%), LM target-lesion revascularization at 5 years was halved with IVUS (5.1% versus 11.6%; *p* = 0.01), and greater post-PCI stent expansion by IVUS was associated with fewer repeat revascularizations [15]. Complementing these cohort and trial data, an analysis of 11,264 unprotected LM PCI procedures from the British Cardiovascular Intervention Society (BCIS) registry showed that the use of intravascular imaging increased from 30.2% to 50.2% over time and it was associated, after propensity matching, with fewer in-hospital coronary complications and MACE (adjusted OR 0.47, 95% CI 0.37–0.59) and significantly lower 30-day and 12-month mortality (OR 0.54, 95% CI 0.43–0.68 and OR 0.66, 95% CI 0.57–0.77, respectively) compared with angiography-guided PCI [16].

The prospective application of such IVUS-optimization criteria in the OPTIVUS-Complex LM cohort (~900 patients)—aiming for a final MSA ≥ 5.0 mm^2^ at the LCx ostium, ≥6.0 mm^2^ at the LAD ostium, ≥7.0 mm^2^ at the polygon of confluence (POC), and ≥8.0 mm^2^ in the proximal LM—yielded a 1-year primary endpoint rate of 13.2%, well below historical PCI performance goals. LM target lesion revascularization was also lower when these prespecified criteria were met [17]. Finally, a recent small single-center randomized trial reported markedly lower 12-month MACE with IVUS-guided LM PCI versus angiography alone (3.3% versus 18.7%) [18]. The results of the ongoing multicenter, randomized OPTIMAL trial will help to better establish the role of PCI guidance in left main PCI [19].

### 2.2. OCT

Optical coherence tomography (OCT) is an intravascular imaging modality that employs near-infrared light to generate high-resolution images, offering an axial resolution of 10–20 μm, markedly superior to that of IVUS. While this enhanced resolution is achieved alongside faster image acquisition times, it comes at the expense of reduced tissue penetration, limited to approximately 3 mm [20]. OCT can be utilized in a variety of clinical settings [21] and is associated with a very low incidence of adverse events, comparable to IVUS [22]. In a large single-center registry comprising over 1100 OCT procedures with over 3000 pullbacks and almost 2500 IVUS procedures with over 5000 pullbacks, a very low rate of adverse events was definitely or possibly associated with the imaging procedure for both modalities (<1%) and without a significant difference between them. Importantly, these adverse effects were scarce, self-limiting after withdrawal of the imaging catheter, or easily treatable in the catheterization laboratory [22]. It should also be noted that OCT imaging requires blood displacement from the lumen, which is mainly achieved by contrast flushing during acquisition. This may lead to an increased volume of contrast in OCT-guided procedures compared to IVUS-guided procedures (~40 mL in the OCTIVUS trial, without a significant difference in contrast-induced nephropathy) [23]. Therefore, IVUS may be preferred in cases that require minimal contrast administration. The principal advantages and limitations of OCT and IVUS are summarized in Table 1 [20,21,22,24].

#### PCI Guidance by OCT in Non-LM Lesions

Evidence for a prognostic benefit of OCT-guided PCI is gradually accumulating. The initial studies of OCT guidance demonstrated the ability of OCT to optimize the procedural outcomes of PCI. DOCTORS and ILUMIEN III demonstrated greater stent expansion and fewer untreated edge dissections/major malapposition versus angiography, without a difference in MACE [25,26]. More recent large RCTs in complex anatomies also including LM bifurcation have shown clinical benefit over angiography: in OCTOBER (complex bifurcations) OCT reduced 2-year MACE (10.1% versus 14.1%; HR ≈ 0.70; *p* = 0.035), and in OCCUPI (complex lesions) OCT lowered 1-year MACE compared with angiography [27,28]. ILUMIEN IV was negative for the primary clinical endpoint but OCT achieved a larger final MSA with and was associated with significantly fewer stent thromboses at 2 years (0.5% versus 1.4%; HR = 0.36; 95% CI 0.14–0.91; *p* = 0.02) [29]; while subgroup analyses in complex lesions showed a significantly lower incidence (3.1% versus 4.9%; HR: 0.63; 95% CI: 0.40–0.99; *p* = 0.04) of “serious MACE” (cardiac death; target-vessel myocardial infarction [MI], or stent thrombosis) [30]. In addition, RENOVATE-COMPLEX PCI—with 1639 “complex PCI” patients randomized 2:1 to intravascular imaging (IVUS or OCT) versus angiography—significantly reduced TVF at a median follow-up of 2.1 years (7.7% versus 12.3%; HR = 0.64; 95% CI 0.45–0.89; *p* = 0.008). OCT was used in the minority of cases (~25%), however the benefit was consistent across the OCT and the IVUS subgroup [31].

Multiple head-to-head trials have compared IVUS with OCT for PCI guidance. In OPINION (*n* = 829), OCT was non-inferior to IVUS for 1-year TVF, with similar late lumen loss/residual stenosis/binary restenosis; interestingly, lumen-based OCT sizing yielded slightly smaller stent diameters versus EEL-based IVUS sizing [32]. ILUMIEN II showed similar degrees of expansion with both techniques, with a possible ~10% overestimation of lumen area by IVUS [33]. In ILUMIEN III (*n* = 450), an OCT strategy using EEL-matching achieved non-inferiority to IVUS for final MSA (5.79 mm^2^ versus 5.89 mm^2^; *p* = 0.01) and fewer major dissections/malapposition, without a difference in MACE [26]. More recently, OCTIVUS (*n* = 2008) demonstrated non-inferiority of OCT versus IVUS for the 1-year composite endpoint (2.5% versus 3.1%; *p* < 0.001 for non-inferiority), confirming that both modalities—when used systematically—deliver comparable clinical outcomes [23]. Taken together, these studies suggest that OCT may offer a reliable alternative to IVUS for PCI guidance in most cases, including complex lesions [34].

## 3. The Role of OCT in LM PCI

### 3.1. Determination of Lesion Significance

The role of OCT for determining lesion significance in LM lesions is not as well defined as the role of IVUS, considering the lack of prospective studies demonstrating prognostic implications of OCT-derived metrics of lesion significance. Initially, there were concerns regarding the use of OCT for guidance in LM PCI due to the limited penetration of OCT and the need for blood displacement, hampering evaluation in large vessels and ostial lesions, respectively. However, pilot studies have demonstrated the potential of imaging left main lesions by OCT, acknowledging the limited depiction of proximal LM lesions and the need for performance of additional pullbacks [35]. Such difficulties in imaging ostial lesions that often require the use of a guide extension catheter [36], may often shift the scale in favor of IVUS in the case of ostial LM assessment. The aforementioned study by Bouki et al. [35] also aimed to determine thresholds for MLA by OCT that can be used to identify the presence of significant lesions based on functional significance by FFR. In a series of 101 patients, an OCT MLA cutoff of 5.38 mm^2^ predicted FFR ≤ 0.80 with 82% sensitivity and 81% specificity [35]. Dato et al. applied arbitrary thresholds to guide the decision of revascularization in LM lesions (MLA ≤ 4 mm^2^ for area stenosis between 50 and 75%, or area stenosis > 75%) [37]. Deferral of revascularization based on the above criteria was safe with similar survival rates between conservative and invasive management (HR: 0.40, 95% CI 0.10–1.61; *p* = 0.20) [37]. Although the wide confidence intervals do not allow the extraction of safe conclusions, these OCT cut-offs contradict the traditional IVUS-derived threshold of MLA < 6 mm^2^, acknowledging OCT’s tendency toward smaller measurements due to superior resolution [10,38,39].

### 3.2. Plaque Assessment

#### 3.2.1. Morphology

**Figure 1:**
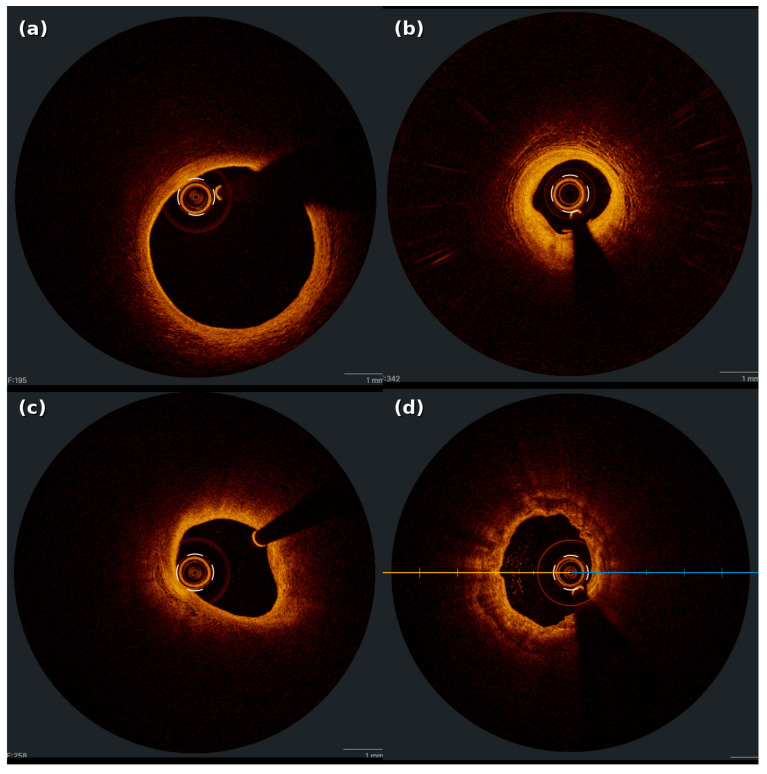
Different plaque types by OCT. (**a**) Normal vessel; (**b**) Fibrous plaque; (**c**) Lipid-rich plaque; (**d**) Fibrocalcific plaque.

The detailed assessment of plaque morphology by OCT plays a critical role in the planning and optimization of PCI procedures. OCT enables precise visualization of plaque composition, including features associated with vulnerability and potential complications. Based on findings from histopathological studies, OCT predominantly categorizes plaques into three types: fibrous, lipid-rich, and calcified (Figure 1) [40,41]. OCT exhibits high diagnostic accuracy for plaque morphology, with sensitivity and specificity of 71–79% and 97–98% for fibrous plaques, 90–94% and 90–92% for lipid-rich plaques, and 95–96% and 97% for calcified plaques, respectively [42]. The identification of thin-cap fibroatheroma (<65 μm fibrous cap thickness) enables risk stratification and procedural planning, particularly in vulnerable plaques associated with increased periprocedural myocardial infarction risk [43,44,45]. This may lead to the adoption of strategies such as cautious predilation with an undersized balloon, direct stenting, and prioritization of complete lesion coverage.

The presence of heavy calcification has been correlated with reduced procedural success during PCI [46], and thus, the identification of calcific burden in LM by OCT should prompt the use of aggressive plaque modification techniques to ensure optimal stent deployment. OCT has been employed to identify parameters predictive of successful modification: calcium arc > 227° and thickness < 0.67 mm predict plaque cracking following rotational atherectomy, while balloon angioplasty alone proves effective only when calcium thickness is <0.24 mm [47]. High calcium burden (thickness > 0.5 mm, length > 5 mm, arc > 180°) correlates with impaired stent expansion [48]. OCT can also distinguish among three types of coronary calcification—superficial, deep, and nodular—each necessitating different interventional strategies. Balloon-based techniques, including scoring or cutting balloons, are generally sufficient for deep calcium, while nodular calcium often requires atherectomy. Superficial calcification can be managed using either atherectomy or intravascular lithotripsy [47].

#### 3.2.2. Landing Zone Determination

**Figure 2:**
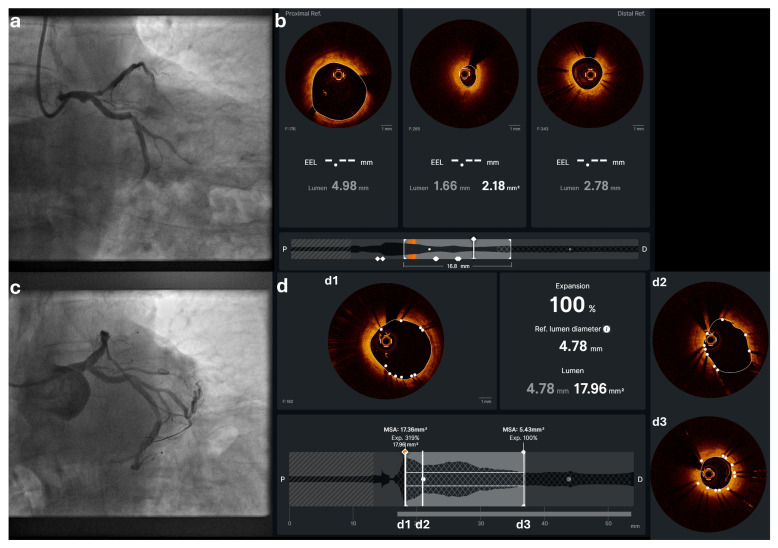
OCT-guided PCI of the LM–LAD: (**a**) baseline angiographic view of the LM–LAD lesion, (**b**) pre-PCI OCT assessment to characterize plaque morphology and assess suitable landing zones to accurately determine vessel dimensions for stent sizing, (**c**) angiographic result post stent implantation, and (**d**) post-PCI OCT demonstrating optimal stent expansion and apposition in the proximal and distal stent edges (**d1**,**d3**), and at the level of the carina (**d2**).

Utilizing L-mode imaging alongside other advanced features, OCT offers precise evaluation of vessel dimensions, structural characteristics, and tissue composition. When planning stent implantation, the optimal landing zones are selected by identifying the largest luminal areas free of significant disease, both proximally and distally to the target lesion (Figure 2). Correct selection of stent length is critical, as incomplete lesion coverage has been linked to increased rates of stent failure and MACE [3]. Importantly, the development of co-registration with angiography allows for the optimization of stent length selection and the minimization of incomplete lesion coverage [49,50].

Ideally, stent landing zones should be located within a healthy vessel segment with visualization of the EEL in the entire circumference of the cross-section. Since this is not always feasible, a minimally diseased non-stenotic segment with predominantly fibrotic tissue (lipid-rich plaque or calcium in less than a quadrant) is usually preferred. A retrospective OCT-based study demonstrated that the presence of lipid-rich plaques adjacent to the stent edges significantly increased the risk of late stent edge restenosis. Furthermore, it was suggested that when facing diffuse disease, selecting landing zones with a lipid arc less than 185° could mitigate the risk of stent edge restenosis [51].

#### 3.2.3. Sizing Principles

Two principal methodologies are employed for stent sizing: a lumen-based and an external elastic lamina (EEL)-based approach [52]. With a lumen-based approach (used in the OPINION trial), stent diameter is selected from the proximal and distal reference lumen diameters adjacent to the lesion [32]. This is practical when the EEL cannot be visualized, but it tends to yield slightly smaller selected stent sizes and post-PCI MSA than EEL-based/IVUS strategies [39,53]. By contrast, an EEL-based approach (evaluated prospectively in ILUMIEN III) involves averaging proximal and distal EEL diameters and rounds down to determine stent diameter. In ILUMIEN III, EEL could be identified in >180° of the vessel circumference in ~84% of cases (identification by core lab in ~95%), and EEL-based OCT achieved stent expansion comparable to IVUS [26,39]. The choice of approach is also subject to practical constraints: in OCT, EEL visualization is reduced in lipid-rich and heavily calcified segments because light attenuation limits media border detection, so a hybrid strategy (EEL where visible, lumen where not) is often necessary [53].

## 4. Principles of OCT Guidance in Bifurcation Lesions

**Figure 3:**
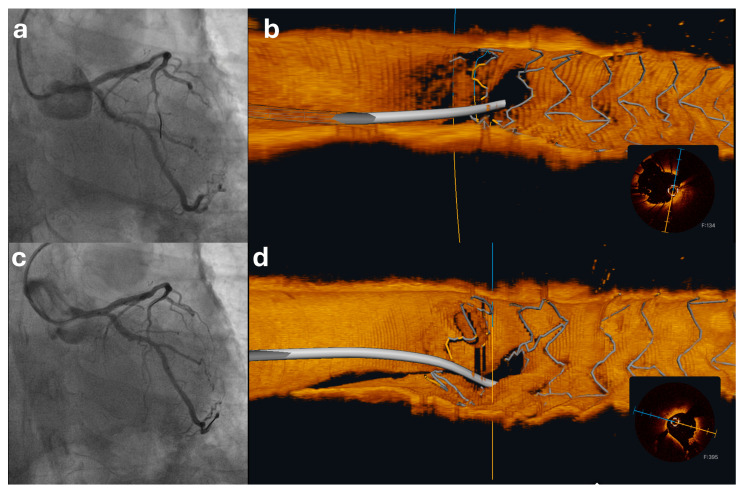
Elective PCI of the proximal left anterior descending artery (LAD) complicated by inadvertent stent displacement toward the left main (LM). (**a**) Angiographic appearance of the left coronary artery immediately after stent implantation with unintentional proximal migration of the stent into the LM was observed. (**b**) Rewiring of the left circumflex (LCx) followed by LM-to-LAD OCT demonstrating that the LCx wire crosses through an appropriate stent cell, with clear visualization of the protruding stent segment in the LM. (**c**) Repeat angiography after kissing balloon dilation of the LM bifurcation (**d**) Corresponding angiography LM-to-LAD OCT confirming adequate stent expansion and apposition with preservation of the LCx ostium.

Lesions at bifurcations, especially those involving the distal left main (LM) coronary artery, present some of the most intricate challenges for PCI. Considering these challenges, these lesions may derive particular benefit from IVI guidance. This was demonstrated in the randomized OCTOBER trial (*n* = 1201; ~19% LM bifurcations) showing that a structured protocol of OCT guidance reduced 2-year MACE compared with angiography (10.1% versus 14.1%; HR 0.70, 95% CI 0.50–0.98; *p* = 0.035), with the prespecified LM subgroup showing concordant benefit (14% versus 19%; HR 0.68, 95% CI 0.46–1.00) and the effect being most pronounced with a one-stent (provisional) strategy (6% versus 12%; HR 0.47, 95% CI 0.24–0.93) [27]. This finding gains particular importance considering findings from the EBC MAIN Trial that failed to demonstrate a benefit for a dual-stent approach over a stepwise provisional strategy with regard to the primary composite endpoint of a composite of death, myocardial infarction, and target lesion revascularization at 12 months, thus suggesting that the stepwise provisional strategy should remain the standard approach in distal LM bifurcation PCI [54]. OCT plays a pivotal role in guiding treatment decisions by evaluating four critical aspects: stenosis at the ostium of the side branch (SB), the bifurcation angle, the length of the disease affecting the proximal SB, and the reference diameter of the distal SB segment. Notably, a bifurcation angle under 50° and a distance of less than 1.70 mm from the proximal bifurcation to the carina tip, in conjunction with significant lipid content in the main vessel (MV) lesion and lipid presence at the bifurcation, have been found to be predictive of potential SB stenosis or occlusion following MV stenting, considering that these morphologies are more susceptible to carina shift [55]. Furthermore, the OCT evaluation should involve both the MV and SB, with stent sizing typically based on the distal MV reference diameter [56]. Moreover, the use of dedicated software may enable the reliable assessment of the SB ostium by a single main vessel pullback, thus aiding the decision for protection of the SB [57]. Importantly, OCT can also be utilized during to procedure for guidance, especially considering the possibility for online 3-dimensional imaging allowing the depiction of complex 3-dimensional structures (Figure 3) [58]. This may allow the assessment of optimal wire position, which is necessary for the optimization of bifurcation intervention, and disclose possible mechanical deformation of the stents that would mandate further correction [59,60]. Intraprocedural assessment may also be used for a change in strategy, such as additional proximal optimization, kissing balloon inflation, or bailout conversion to two-stent techniques. Consistent with these principles, OCTOBER operationalized OCT guidance through predefined procedural targets—complete lesion coverage without significant edge disease, adequate stent expansion, and absence of malapposition—and systematic confirmation of optimal SB wiring and final result (including kissing inflation and proximal optimization technique when indicated) in bifurcations, including distal LM [27,61]. After stent implantation, OCT assessment can reveal factors indicative of poor long-term prognosis, such as edge dissection, stent malapposition, and underexpansion, providing the opportunity for optimization of the result.

## 5. Studies of OCT Guidance in LM PCI

Several trials have investigated the potential of OCT guidance for LM PCI. The LEMON Study, a multicenter prospective trial, assessed the safety, feasibility, and impact of OCT-guided PCI for mid/distal LM lesions in 70 patients. The study found that 86% of the patients achieved a combination of less than 50% residual angiographic stenosis, TIMI 3 flow in all branches, and adequate stent expansion, with 26% of cases requiring a modification of the initial strategy based on OCT findings. These results led to a 98.6% rate of freedom from major adverse cardiovascular events (MACE) at one year, supporting the feasibility of OCT-guided LM PCI [62]. Similarly, in a study by Agrawal et al., 110 stent implantations (including LM lesions) in 100 consecutive patients were reviewed, with OCT performed after the operator deemed the stent optimally deployed based on coronary angiography. Interestingly, strut malapposition was detected in 74.5% of cases, with the most common mechanisms being localized lumen enlargement, followed by stent undersizing (46.3%), strut underexpansion (29.3%), stent deployment issues (18.2%), and vessel asymmetry (9.7%) [63]. These findings highlight that OCT can identify malapposition and underexpansion more effectively than coronary angiography alone and can guide physicians toward the most appropriate strategy to address these issues.

In this context, contemporary real-world data from a single-center high-volume LM registry (2013–2024; *n* = 221) reported that OCT-guided unprotected LM PCI (13.1% of cases) was associated with a higher unadjusted survival probability versus angiography guidance (Kaplan–Meier log-rank *p* = 0.034) and a trend towards independent reduction in mortality on multivariable analysis (adjusted HR 0.37; *p* = 0.063). Procedurally, OCT prompted intraprocedural optimization frequently (additional therapy after the first OCT run in ~48%) and was associated with more strut recross/rewiring (42.3% versus 23.0%; *p* = 0.034), consistent with the modality’s ability to detect actionable issues before case completion [64]. Complementary long-term OCT follow-up after primary unprotected LM PCI (*n* = 15; mean 1580 ± 1260 days) demonstrated persistent healing deficits concentrated in the LM and POC segments—higher malapposed and lower covered strut proportions than the distal main branch—and substantial late pathology (neoatherosclerosis 33%, repeat PCI 33%). These observations underscore why meticulous OCT-guided optimization at index LM PCI (minimizing underexpansion and malapposition) may be critical for durable outcomes [65]. However, further studies are needed to evaluate the long-term clinical outcomes associated with OCT-guided stenting in LM lesions.

## 6. OCT Versus IVUS in Left Main PCI

Although evidence regarding the application of OCT in LM coronary interventions remains relatively sparse, comparisons between OCT and IVUS in this context have generated significant interest, yet they remain only partially investigated. In a prospective study, Fujino et al. directly compared the two modalities, demonstrating that OCT and IVUS yielded comparable results for mean lumen and stent area measurements (11.24 ± 2.66 mm^2^ versus 10.85 ± 2.47 mm^2^; *p* = 0.13, and 10.44 ± 2.33 mm^2^ versus 10.49 ± 2.32 mm^2^; *p* = 0.82, respectively). However, OCT exhibited a superior ability to detect stent malapposition (malapposition area 0.43 ± 0.51 mm^2^ versus 0.12 ± 0.36 mm^2^; *p* < 0.001) and distal edge dissections (30.3% versus 6.1%; *p* = 0.011). Importantly, OCT maintained a favorable safety profile comparable to that of IVUS, with no adverse events such as ST-segment changes, vessel occlusions, dissections, coronary spasms, or slow-flow phenomena being reported [66].

Further supporting this, the multicenter, retrospective ROCK I study by Cortese et al. evaluated OCT-guided versus standard imaging-guided stenting (angiography with or without IVUS at operator discretion) in distal LM PCI. The results revealed that late lumen loss at six months was lower in the OCT group, particularly in the distal stent segment (0.03 ± 0.45 mm versus 0.24 ± 0.53 mm; *p* = 0.025), although the reduction in the proximal segment did not reach statistical significance (0.12 ± 0.41 mm versus 0.26 ± 0.52 mm; *p* = 0.10). Additionally, OCT guidance was associated with a decrease in percent diameter stenosis (14 ± 9% versus 19 ± 16%; *p* = 0.05) and restenosis rates (3.5% versus 12.9%; *p* = 0.03) [67]. Expanding on these findings, the multicenter, international ROCK II study enrolled 730 patients undergoing distal LM stenting and found no significant differences in target lesion failure (TLF) —a composite of cardiac death, target vessel myocardial infarction, and target lesion revascularization—between the OCT and IVUS groups at one-year follow-up (*p* = 0.26). Even after propensity-score matching, TLF rates remained similar (7% versus 6%), with no notable differences observed in the individual components of the composite outcome. Notably, OCT continued to outperform IVUS in the detection of acute stent malapposition and residual edge dissection (10% versus 4%; *p* = 0.04; 9.7% versus 5.1%; *p* = 0.04, respectively) [68].

Another investigation involving 331 patients reported comparable rates of restenosis (15.2% versus 9.4%; *p* = 0.39) and major adverse cardiac events (MACE) (7.0% versus 7.4%; *p* = 0.98) between OCT- and IVUS-guided interventions, suggesting similar clinical effectiveness between the two modalities [69]. A sub-analysis of the OCTIVUS study compared OCT guidance with IVUS in patients undergoing stenting for LM coronary artery lesions. In this trial, there were no significant differences in TLF rates at one year, with similar clinical outcomes observed between the two groups. These findings suggest that while OCT offers superior technical outcomes in detecting stent malapposition and dissections in LM lesions, these benefits do not translate into long-term clinical advantages in terms of restenosis or MACE [23].

Consistent with the neutral head-to-head signal, a 2025 systematic review and meta-analysis of seven randomized trials reported that intravascular imaging guidance—with either OCT or IVUS—reduces target vessel failure compared with angiography in unprotected LM PCI (*n* = 1107; RR 0.55, 95% CI 0.36–0.84; NNT = 11) and in bifurcation PCI (*n* = 2494; RR 0.70, 95% CI 0.53–0.92; NNT = 27). Crucially, the analysis did not adjudicate device-specific differences between OCT and IVUS; thus, while IVI is beneficial in LM PCI, superiority of one modality over the other cannot be inferred [70].

While these emerging data are promising, they underline the urgent necessity for larger, randomized controlled trials to further establish the role of OCT in LM disease, with the potential to enhance both procedural success and long-term clinical outcomes. An overview of dedicated OCT-based studies in unprotected LM disease is presented in Table 2, while comparative OCT versus IVUS or angiography data in LM PCI are summarized in Table 3.

## 7. Conclusions

Left main coronary artery disease remains a high-risk disease entity in which percutaneous revascularization is now an established alternative to surgery in carefully selected patients. In this complex anatomical and prognostically important setting, intravascular imaging has become a cornerstone of contemporary practice, allowing more precise lesion assessment, optimal device selection, and systematic stent optimization. While IVUS continues to represent the reference standard due to its longer track record and broader evidence base, OCT has matured into a robust imaging modality with unique strengths that are particularly relevant for LM PCI.

OCT provides high-resolution characterization of plaque morphology and calcium burden, facilitates accurate landing-zone selection and stent sizing (including EEL-based strategies in suitable segments), and offers unparalleled sensitivity for detecting underexpansion, malapposition, and edge pathology. In distal LM bifurcations, 3-dimensional reconstructions and detailed carina/side-branch assessment further support procedural planning, guide SB protection and rewiring, and enable iterative optimization of the final result. Observational LM series and trials in complex lesions have demonstrated that OCT-guided strategies are safe and feasible; frequently lead to intraprocedural strategy modification or additional optimization; and achieve comparable clinical outcomes to IVUS, while consistently improving the detection of actionable mechanical issues.

Despite these encouraging data, dedicated, adequately powered randomized trials focused on LM disease are still required to define standardized OCT-based optimization targets and to clarify whether the modality’s superior technical performance translates into incremental long-term prognostic benefit. Until such evidence is available, the choice between OCT and IVUS should be individualized, taking into account vessel size, ostial anatomy, contrast load, and local expertise. Within this framework, the growing body of LM-specific experience provides a strong rationale to actively consider OCT as a preferred intravascular imaging option in anatomically suitable LM interventions, particularly when meticulous bifurcation optimization and detailed stent assessment are critical to securing durable outcomes.

## Figures and Tables

**Table 1:** Advantages and disadvantages of OCT and IVUS in LM intervention guidance.

OCT	IVUS
**Advantages**	
High spatial resolution (10–20 μm axial, 20–40 μm lateral)	Better penetration
Better tissue characterization (calcium)	More frequent EEL identification for vessel-based sizing
Thrombus identification	Allows better quantification of plaque volume
Assessment of stent edge dissection, strut coverage, and malapposition	Accurate evaluation of stent area and stent expansion
Better guidance for SB rewiring (of particular interest in distal LM)	Extensive clinical experience and more available randomized evidence, including the LM setting
Easier to interpret	Valuable guide in CTO procedures
**Disadvantages**	
Limited penetration (impeding large vessel evaluation, such as LM)	Poor tissue characterization
Need for contrast media injection	Difficult thrombus identification
Need for flushing to remove blood (difficulty in evaluating LM ostium)	Limited identification of strut coverage, strut malapposition
Less prognostic data in PCI guidance	Difficult to interpret

**Table 2:** Dedicated OCT-based assessment and/or guidance in unprotected left main disease.

Study (Year)	LM Population/Setting	Design/OCT Strategy	Key LM-Related OCT Findings
Bouki et al., 2021 [35]	Angiographically intermediate (20–70%) non-ostial LM stenosis undergoing FFR and OCT.	Prospective single-center study; OCT measurements (MLA, MLD, %AS) correlated with FFR to define LM-specific functional cut-offs.	FFR ≤ 0.80 was present in 44.3% of analyzable lesions. An OCT-derived MLA ≤ 5.38 mm^2^ predicted functionally significant LM stenosis with AUC 0.90, sensitivity 82% and specificity 81%, supporting a LM MLA threshold slightly lower than the traditional IVUS-derived 6 mm^2^.
Dato et al., 2017 [37]	Angiographically intermediate unprotected LM lesions managed with arbitrary OCT criteria.	Retrospective cohort; treatment guided by a hybrid algorithm combining OCT-derived MLA and %AS to decide on LM PCI versus deferral.	At 18-month follow-up, survival did not differ between revascularized and deferred lesions (HR 0.40; 95% CI 0.10–1.61). The hybrid strategy used OCT cut-offs of AS > 75% or MLA ≤ 4.0 mm^2^ when AS was 50–75% to trigger LM PCI, while lesions above these thresholds were safely deferred.
LEMON (Amabile et al., 2021) [62]	Patients undergoing PCI for unprotected LM disease in a multicentre OCT-guided registry.	Non-randomized prospective study; systematic pre- and post-PCI OCT used to optimize LM stent sizing, expansion and bifurcation strategy.	Composite procedural success was achieved in 86%, and freedom from MACE at 1 year was 98.6%. OCT frequently prompted further post-dilatation and treatment of edge dissections or malapposition for optimization.
Lazar et al., 2025 [64]	Real-world cohort of patients treated with PCI for significant unprotected LM disease.	Retrospective single-center registry comparing OCT-guided versus angiography-guided LM PCI; at least one OCT run required in the OCT arm.	Among 221 patients (13.1% OCT-guided), over a median follow-up of ≈30 months, OCT guidance was associated with higher survival (log-rank *p* = 0.034). In multivariable analysis, OCT was associated with a trend for ~63% relative reduction in all-cause mortality (HR 0.37; *p* ≈ 0.06).
Mehmedbegovic et al., 2024 [65]	Patients with previous primary PCI for culprit unprotected LM lesions undergoing very late OCT follow-up of DES.	Single-center observational study; long-term (≈4.3 years) OCT assessment of DES healing in LM, polygon of confluence and distal main branch segments.	High-quality OCT images were obtained in 93.3% of cases. Malapposed struts were more frequent and coverage was lower in LM/POC versus distal segments (malapposition ≈ 11–13% versus 0.3%; covered struts ≈ 82–84% versus 92%). Neoatherosclerosis was present in 33.3% and restenotic neointimal hyperplasia in 13.3%, with repeat PCI required in one-third of patients.

**Table 3:** Comparative OCT versus IVUS or angiography in unprotected left main PCI.

Study (Year)	Comparator/LM Population	Design	Main LM-Related Outcomes
Fujino et al., 2013 [66]	Patients undergoing PCI for unprotected, non-ostial LM disease imaged with both OCT and IVUS.	Prospective comparative imaging study with paired OCT–IVUS measurements of pre- and post-stent LM lumen and stent dimensions.	OCT and IVUS yielded closely matching LM lumen and stent areas (mean MLA around 11 mm^2^ with both modalities), with excellent correlation. OCT provided better assessment of strut apposition and edge pathology, and no OCT-related LM complications were reported.
ROCK I (Cortese et al., 2020) [67]	Patients undergoing distal LM bifurcation PCI; OCT-guided versus angiography-guided PCI (with selective IVUS use).	Retrospective multicentre study with angiographic follow-up at 6 months to assess late lumen loss in distal LM and main branch segments.	Late lumen loss in the distal LM/LAD–Cx segment was significantly lower with OCT guidance (0.03 ± 0.45 mm versus 0.24 ± 0.53 mm; *p* = 0.025), while overall clinical event rates at follow-up were low and similar between groups.
ROCK II (Cortese et al., 2022) [68]	Distal LM bifurcation PCI; OCT- or IVUS-guided PCI versus angiography-guided PCI in routine clinical practice.	Retrospective multicentre registry with 1-year clinical follow-up comparing imaging-guided versus angiography-only LM PCI and, within the imaging arm, OCT versus IVUS.	Imaging guidance (OCT/IVUS pooled) reduced 1-year target lesion failure compared with angiography alone (12.7% versus 21.2%; *p* = 0.039). Within the imaging-guided group, rates of cardiac death, target-vessel MI and TLR were similar between OCT and IVUS (*p* = 0.26).
Miura et al., 2021 [69]	LM bifurcation PCI guided by 3D-OCT versus IVUS.	Retrospective cohort with systematic use of 3D-OCT or IVUS for LM bifurcation PCI and 8-month angiographic and clinical follow-up.	Clinical outcomes were similar between modalities: MACE 7.0% (3D-OCT) versus 7.4% (IVUS; *p* = 0.98) and angiographic restenosis 15.2% versus 9.4% (*p* = 0.387).
OCTIVUS (Kang et al., 2023) [23]	Patients with complex coronary disease, including LM lesions, randomized to OCT-guided versus IVUS-guided PCI.	Multicenter randomized trial; prespecified 2-year analysis with subgroup evaluation according to the presence or absence of LM disease.	In the overall trial, target vessel failure at 2 years occurred in 6.5% of OCT-guided versus 7.4% of IVUS-guided cases (HR 0.87; 95% CI 0.59–1.29). Subgroup analyses showed no significant interaction for left main involvement (*p* for interaction > 0.1).
DOCTORS-LM (NCT04391413)	Patients undergoing PCI for unprotected LM; OCT-guided versus angiography-guided PCI.	Prospective randomized study extending the DOCTORS concept to LM disease; designed to compare functional and clinical outcomes with OCT-guided versus angiography-guided LM PCI.	Trial recruitment and follow-up are ongoing.

## Data Availability

Not applicable. No new data were created or analyzed in this study. Data sharing is not applicable to this article.

## Abbreviations

The following abbreviations are used in this manuscript:

3D-OCT	3-dimensional optical coherence tomography
AS	area stenosis
AUC	area under the curve
CABG	coronary artery bypass grafting
CAD	coronary artery disease
CI	confidence intervals
CTO	chronic total occlusion
DES	drug-eluting stents
EEL	external elastic lamina
FFR	fractional flow reserve
HR	hazard ratio
IVI	intravascular imaging
IVUS	intravascular ultrasound
LAD	left anterior descending
LCx	left circumflex
LM	Left main
MACE	major adverse cardiac events
MI	myocardial infarction
MLA	minimum lumen area
MLD	minimum lumen diameter
MSA	minimum stent area
MV	main vessel
NNT	number needed to treat
OCT	Optical coherence tomography
OR	odds ratio
PCI	percutaneous coronary intervention
POC	polygon of confluence
RR	relative risk
SB	side branch
ST	stent thrombosis
TLF	target lesion failure
TLR	target lesion revascularization
TVF	target vessel failure
